# Is it time to re-think FAST? A systematic review and meta-analysis of Contrast-Enhanced Ultrasound (CEUS) and conventional ultrasound for initial assessment of abdominal trauma

**DOI:** 10.1186/s12873-023-00771-4

**Published:** 2023-01-27

**Authors:** Bayu Sutarjono, Matthew Kessel, Dorian Alexander, Ekjot Grewal

**Affiliations:** Department of Emergency Medicine, Brookdale University Hospital and Medical Center, 1 Brookdale Plaza, Brooklyn, NY 11212 USA

**Keywords:** Contrast-enhanced ultrasound, CEUS, Trauma, Emergency medicine

## Abstract

**Background:**

The Focused Assessment with Sonography for Trauma (FAST) examination using conventional ultrasound has limited utility for detecting solid organ injury. Therefore, this systematic review and meta-analysis compares the performance of contrast-enhanced ultrasound (CEUS) to conventional ultrasound when used as the initial assessment for abdominal trauma prior to computed tomography (CT) imaging.

**Methods:**

A systematic literature search of major databases was conducted of human studies investigating the diagnostic accuracy of conventional ultrasound and CEUS occurring prior to CT imaging for abdominal trauma. The study followed the PRISMA (Preferred Reporting Items for Systematic Reviews and Meta-Analyses) statement. The quality of studies was evaluated using the QUADAS-2 (Quality Assessment of Diagnostic Accuracy Studies 2) tool. Paired pooled sensitivity and specificity between conventional ultrasound and CEUS were compared using data extracted from the eligible studies. Diagnostic odds ratio, number needed to diagnose values, and likelihood ratios were also determined.

**Results:**

Ten studies were included. More than half of the included studies demonstrated low risk of bias. Using McNemar’s test to assess for paired binary observations, we found that CEUS had statistically higher sensitivity (0.933 vs. 0.559; two-tailed, *P* < 0.001) and specificity (0.995 vs. 0.979; two-tailed, *P* < 0.001) than conventional ultrasound in the setting of abdominal trauma, respectively. When divided into particular findings of clinical interest, CEUS had statistically higher sensitivity than conventional ultrasound in screening for active bleeding and injuries to all abdominal solid organs. CEUS also had superior diagnostic odds ratios, number needed to diagnose values, and likelihood ratios than conventional ultrasound.

**Conclusion:**

The diagnostic value of CEUS was higher than that of conventional ultrasound for differentiating traumatic abdominal injuries when used as the initial assessment in the emergency department.

**Supplementary Information:**

The online version contains supplementary material available at 10.1186/s12873-023-00771-4.

## Background

The Focused Assessment with Sonography for Trauma (FAST) examination using conventional ultrasound has been an integral part of the evaluation of trauma patients for over 20 years [1]. While the FAST is an extremely useful bedside tool for ruling in intraabdominal free fluid in the trauma resuscitation setting, it has limited utility for detecting solid organ injury, particularly in the absence of intraperitoneal free fluid [2].

To date, there are numerous studies that investigated whether the use of contrast-enhanced agents with ultrasound, also known as contrast-enhanced ultrasound (CEUS), improved the sensitivity and specificity for detecting abdominal traumatic lesions. Utilizing small intravenous boluses of inert gas-filled microbubbles with a phospholipid shell, acute solid organ lesions can be depicted in real time through all the vascular phases [3, 4]. Furthermore, these agents are well tolerated in patients, particularly those with renal insufficiency, hypotension, or shock [5–7].

Currently, there are multiple studies that report the benefits of using CEUS for the identification of abdominal injuries following trauma [8–10], however none directly compare its accuracy to conventional ultrasound nor its effectiveness when utilized with the FAST exam during the initial trauma assessment. Therefore, the objective of this systematic review and meta-analysis was to compare the performance of conventional ultrasound and CEUS when used as the initial assessment for abdominal trauma, whereby all sonographic examinations have been completed prior to computed tomography (CT) imaging.

## Methods

We performed a systematic review and meta-analysis of studies that compared the performance of CEUS to conventional ultrasound when used as the initial assessment for abdominal trauma prior to CT imaging. This study followed the guidelines in the “Cochrane Handbook for Systematic Reviews of Diagnostic Test Accuracy” [11]. This study was not registered.

### Eligibility criteria

Studies were considered eligible for this systematic review and meta-analysis if they fulfilled the following criteria: 1) human studies investigating the diagnostic accuracy of conventional ultrasound, CEUS, and CT for abdominal trauma, with the reference standard clearly defined; 2) both conventional ultrasound and CEUS diagnostic tests must have been performed prior to assessments by CT scan; 3) all diagnostic tests must have been completed within 3 hours of the patient’s presentation to the emergency department; and 4) both prospective and retrospective studies were eligible. We excluded studies when they met one of the following criteria: 1) experimentation with animals; 2) reviews, commentary, and case reports; and 3) non-traumatic conditions.

### Search strategy

A standardized search was done in PubMed, OVID MEDLINE, Embase, and Web of Science, using the following search terms: (“contrast enhanced ultrasound” OR “contrast enhanced sonography” OR CES OR CEUS) AND trauma. The search was done on January 1, 2022 with no language restrictions.

### Study selection

Two authors screened and selected studies independently based on the criteria described above, with disagreements resolved by consensus together with a third author. Studies identified from different databases were de-duplicated after screening. Articles that passed the initial screening were reviewed for the full text. Studies with data available on true negative, true positive, false negative, and false positive results were included for the meta-analysis. This study followed the Preferred Reporting Items for a Systematic Review and Meta-analysis of Diagnostic Test Accuracy (PRISMA) [12]. The checklist can be found in the Supplemental Material.

### Data collection process and data items

For the included studies, individual data of sample size, number of true negative, true positive, false negative, and false positive results per imaging modality in each study were extracted. If only partial information was available, outcomes were calculated using results from CT imaging as reference standard. Based upon how findings were presented in the included studies, datasets were nominally categorized according to identified injured organs or findings. These organs included the 1) liver, 2) kidneys and adrenals, 3) spleen, and 4) pancreas. The findings included 5) abdominal free fluid, 6) active bleeding, 7) any solid organ injury, or 8) a combination of solid organ injury, abdominal free fluid and/or active bleeding (composite). Dichotomous findings of positive or negative for each were compared against CT findings as the gold standard.

Information on location, study design, setting, mechanism and site of injury, contrast and dose, technical aspects of the ultrasound machine, and characteristics of the sonographer and retrospective reviewer of ultrasound clips were also retrieved.

### Risk of bias

The quality of each study was appraised with the Quality Assessment of Diagnostic Accuracy Studies 2 (QUADAS-2) tool, structured into patient selection, index test, reference standard, and flow and timing, structured as a list of 13 items and and qualified as “yes,” “no,” or “unclear” for an individual study. Each domain was evaluated for the risk of bias and the first three in terms of applicability. The answers were used to judge whether the risk of bias and concern for the applicability of the research is low, high, or unclear. Two reviewers independently judged the quality of each study, with disagreements resolved by consensus with additional input from a third.

### Synthesis of results

Subgroup analyses for all categories of abdominal injury were conducted using only studies that provided paired data for both conventional ultrasound and CEUS. Articles that did not provide data for both modalities were excluded from this portion of the study. Sensitivity, specificity, diagnostic odds ratios, number needed to diagnose, and likelihood ratios with the associated 95% confidence intervals were calculated from true negative, true positive, false negative, and false positive cases with a 0.5 continuity correction for zero events. Equations are found in the Supplemental Material. Since the patients underwent both conventional ultrasound and CEUS consecutively prior to obtaining CT imaging, McNemar’s chi-square test was chosen to compare paired pooled sensitivity and specificity. All *P* values were two-sided, and any *P* value < 0.05 was considered statistically significant. Forest plots were generated for individual studies according to solid organ injury or finding along with summary estimates for each category and overall estimates. Heterogeneity was assessed, whereby *P* < 0.05 for Cochran’s Q and Higgin’s I^2^ > 0.500 indicate significant heterogeneity. Since variability among studies was not only due to sampling error, but also to variability in the population of effects, the random effects model using the inverse variance method was used if heterogeneity is high, which was determined by comparing the Cochrane Q to the critical value for its respective degree of freedom as found in a chi-square distribution, and subsequently I^2^ > 0.500 using the fixed effect model. Summary receiver operating characteristics (SROC) curve plotting sensitivity (true positive rate) against 1-specificity (false positive rate) was generated. This was created by plotting the true positive rate against the false positive rate at various threshold values as a scatter plot, after which the area under the curve (AUC) can be calculated. The AUC served as proxy for diagnostic accuracy, whereby AUC > 0.900 indicate excellent diagnostic accuracy. All statistical analyses were performed using Microsoft Excel (version 2021).

## Results

**Fig. 1:**
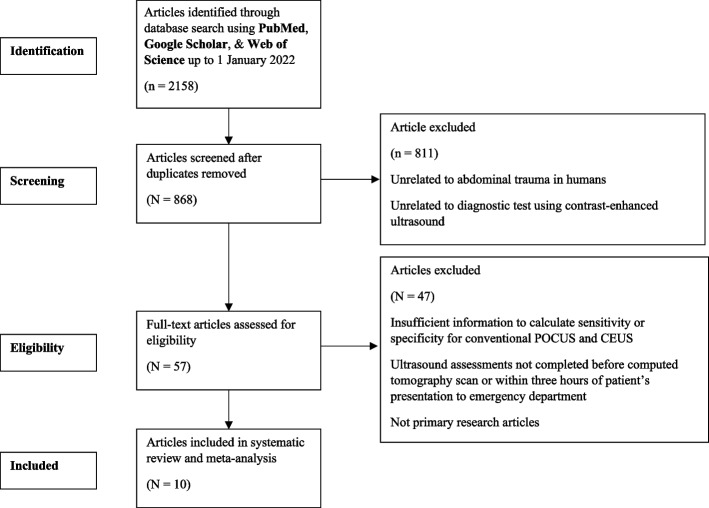
Study profile

Eight hundred sixty-eight studies were identified in our search. After assessing the titles and abstracts, 57 full texts were screened, as shown in Fig. 1. On the basis of our selection criteria, 47 of those studies were excluded, two of which consisted of CEUS studies with no conventional ultrasound diagnostic tests performed [13, 14]. Therefore, 10 studies [15–24] met our inclusion criteria for a total of 1359 patients.

All studies were published after the year 2000 and four studies were published after 2010 [18, 19, 22, 24]. Nine studies were conducted in Europe, predominantly in Italy [15–17, 19–24], while only one study took place in Asia [18]. Only four studies were conducted prospectively [15, 17, 21, 23], with two designed as multi-center studies [17, 18]. Nine studies out of ten reported using SonoVue contrast agent (sulfur hexafluoride lipid Type A microspheres; Bracco diagnostics Inc.), also known as Lumason in the United States, with doses ranging from 1.2 mL to 4.8 mL [15–23]. Scanning time of the entire abdomen ranged from 1 minute to 6 minutes in total. The participants of eight studies were comprised of adults [15, 16, 18, 20–24], while two studies involved the pediatric population [17, 19], one of which enrolled individuals exclusively under the age of 17 years [19]. All studies enrolled patients experiencing blunt abdominal trauma, with two studies including penetrating abdominal trauma as well [16, 17].

The most common organ investigated was the liver [16, 17, 19, 20, 22–24], while the least common was the pancreas [18, 24]. Less than half of the studies presented data of a variety of injury types within the abdomen, including signs of solid organ injuries, the existence of abdominal free fluid, and the presence of active bleeding [17, 19, 23, 24]. On the other hand, the focus of five studies was a single targeted organ or injury [15, 16, 18, 20, 21]. Evidence of hypoperfused regions were visualized as hypoechoic and/or hypodense well-defined areas of solid organs, while contusions were presented as areas with subtle and inhomogeneous echogenicity without mass effect or parenchymal vessel displacement. Hypoechoic linear or branched bands on organ surfaces were interpreted as lacerations, whereas hematomas were described as poorly delineated inhomogeneous collections within the parenchyma. Abdominal free fluid was defined as anechoic intraperitoneal fluid. Parenchymal active bleeding was identified with CEUS as microbubbles within lesions of a solid organ or focal extravasation of microbubbles outside of a lacerated organ. Examinations by conventional ultrasound and CEUS were completed within 1 hour of patient arrival in the emergency department in the majority of studies [15, 17–24], with the remaining study completing the sonographic examinations within 3 hours [16]. Characteristics of included studies are shown in Table 1.

**Table 1:** Characteristics of included studies

Author	Study design, setting	N	Mechanism of trauma; injury	Mean Age	Contrast, dose; protocol (scanning time)	Transducer; mechanical index	Sonographer; ultrasound image interpreter
Catalano [15]	Sing. Pro., ED.	120	Blunt; Spleen Abd. Fluid Act. Bleed	28	Sonovue, 4.8 mL; Left upper quad. (3–4 min total)	2.5–5 MHz or 5.5–10 MHz curved; 0.06–0.07	Radiologist on duty; Two radiologists, third for disagreements
Catalano [16]	Sing. Retro., ED.	87	Blunt or Pen.; Liver Abd. fluid	33	SonoVue, 2.4–4.8 mL; 1. Left kidney 2. Right kidney & liver 3. Spleen (1–5 min total)	3.5 MHz curved; 0.06–0.08	Radiologist on duty; Two radiologists, third for disagreements
Catalano [17]	Mult. Pro., ED.	156	Blunt or Pen.; Liver Kidneys & adrenal gl. Spleen Comp.	39	SonoVue, 2.4 mL (two doses); 1. Right sided organs (right kidney, adrenal, liver) (1–3 min) 2. Left sided organs (left kidney, adrenal, pancreas, spleen) (3–4 min)	2.5 MHz or 1–5 MHz curved; 0.05–0.10	Radiologist on duty; Same
Lv [18]	Mult. Retro., ED.	22	Blunt; Pancreas	29	SonoVue, 2.5 mL/kg; Liver & spleen (NA min)	2–4.5 MHz or 1–5 MHz curved; Low	Two ultrasound specialists (5 years exp.); Same
Menichini [19]	Sing. Retro., ED.	73	Blunt; Liver Kidneys & adrenal gl. Spleen Act. Bleed Solid org. Comp.	9	SonoVue, 1.2 mL (two doses); 1. Right & left kidneys 2. Liver 3. Pancreas 4. Spleen (3 min total)	Curved and linear; Low	Three radiologists (10+ years exp.); Same
Miele [20]	Sing. Retro., ED.	203	Blunt; Liver	36	SonoVue, 2 mL (two doses); Liver (10–15 min total)	Curved; 0.2	NA; NA
Regine [21]	Sing. Pros., ED.	277	Blunt; Kidneys & adrenal gl.	NA	SonoVue, 2.4 mL; 1. Liver 2. Spleen, 3. Kidneys, 4. Supra- & submesocolic peritoneal recesses (NA min)	NA; Low	NA; NA
Sessa [22]	Sing. Retro., ED.	256	Blunt; Liver Kidneys & adrenal gl. Spleen Abd. Fluid Act. Bleed	41	SonoVue, 2.4 mL (two doses); 1. Right sided organs (right kidney, liver) (1–3 min) 2. Left sided organs (left kidney, spleen) (3–4 min)	4 MHz curved; 0.15–0.19	Multiple radiologists (5+ years exp. In emergency radiology & expertise in trauma imaging); NA
Valentino [23]	Sing. Pro., ED.	32	Blunt; Liver Kidneys & adrenal gl. Spleen Abd. Fluid Solid org. Comp.	38	SonoVue, 2.4 mL (two doses); 1. Left upper quad. (left kidney & adrenal gland, spleen) 2. Right upper quad. (right kidney & adrenal gland, liver, pancreas) (4–6 min total)	3.5 MHz or 2–5 MHz curved; Low	Multiple sonographers (5+ years exp.); Independent expert sonography and radiologist
Valentino [24]	Sing. Retro., ED.	133	Blunt; Liver Kidneys & adrenal gl. Pancreas Spleen Abd. Fluid Solid org. Comp.	40	NA, 2.4 mL (two doses); 1. Right upper quad. (right kidney, liver, pancreas) 2. Left upper quad. (left kidney, spleen) (NA min)	2–5 MHz curved; Low	One radiologist; Two radiologists not involved with examination

*Abbreviations*: *Sing*. single-center, *Mult*. multi-center, *Pro*. prospective, *Retro*. retrospective, *ED*. emergency department, *Pen*. penetrating, *Exp*. experience, *NA* not available, *Adrenal gl*. adrenal glands, *Abd*. *Fluid* abdominal free fluid, *Act*. *Bleed* active bleeding, *Solid org*. Any solid organ injury, *Comp*. solid organ injury, abdominal free fluid, and/or active bleeding, *Quad*. quadrant

**Fig. 2:**
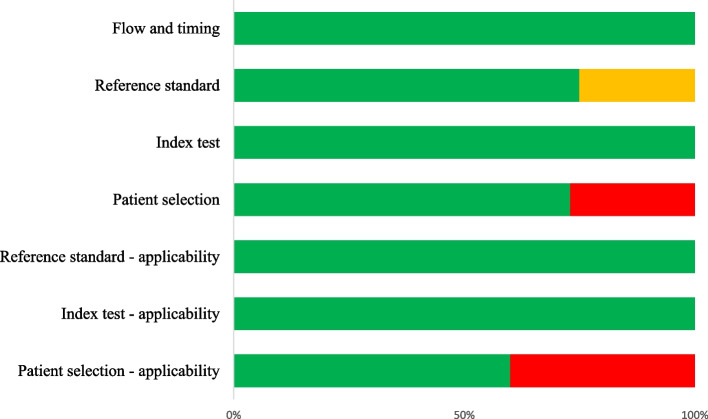
Quality assessment of diagnostic accuracy studies 2 (QUADAS-2) finding per domain for included studies in the systematic review. Green represents low level of bias, red represents high level of bias, while orange represents unclear level of bias

Table 2 depicts the risk of bias assessment using the QUADAS-2 tool, with visual representation in Fig. 2. Less than half of all studies (4) had either high risk of patient selection bias due to non-consecutive or non-random selection of patients. Selection of patients for CEUS examination was determined by a qualified physician conducting the conventional ultrasound baseline assessment [15, 16, 23, 24]. All studies had low risk of index test bias and flow and timing bias. However, it was unclear whether there was risk of reference standard bias in half of all studies (5) as it was not reported whether CT images were interpreted by blinded individuals [16, 20–22, 24]. It is important to note that five of the ten studies selected for the systematic review were authored by two individuals [15–17, 23, 24], both of whom conducted retrospective studies that may have reused a small portion of the patient population from earlier prospective studies [16, 23]. Authors of these studies were unable to be contacted for clarification.

**Table 2:** Risk of bias assessment performed with the Quality of Assessment of Diagnostic Accuracy Studies-2 (QUADAS-2) tool

Risk of bias											Applicability concerns		
	Domain 1: Patient selection			Domain 2: Index text		Domain 3: Reference standard		Domain 4: Flow and timing			Patient selection	Index test	Reference standard
	1. Was a consecutive or random sample of patients enrolled?	2. Was a a case-control design avoided?	3. Did the study avoid inappropriate exclusions?	1. Were the index test results interpreted without knowledge of the reference standard?	2. If a threshold was used, was it pre-specified?	1. Is the reference standard likely to correctly classify the target condition?	2. Were the reference standard results interpreted without knowledge of the results of the index?	1. Was there an appropriate interval between index test and reference standard?	2. Did all patients receive the same reference standard?	3. Were all patients included in the analysis?			
Catalano [15]	No	No	Yes	Yes	Yes	Yes	Yes	Yes	Yes	Yes	High	Low	Yes
Catalano [16]	No	No	Yes	Yes	Yes	Yes	Unclear	Yes	Yes	Yes	High	Low	Yes
Catalano [17]	Yes	Yes	Yes	Yes	Yes	Yes	Yes	Yes	Yes	Yes	Low	Low	Yes
Lv [18]	Yes	Yes	Yes	Yes	Yes	Yes	Yes	Yes	Yes	Yes	Low	Low	Yes
Menichini [19]	Yes	Yes	Yes	Yes	Yes	Yes	Yes	Yes	Yes	Yes	Low	Low	Yes
Miele [20]	Yes	Yes	Yes	Yes	Yes	Yes	Unclear	Yes	Yes	Yes	Low	Low	Yes
Regine [21]	Yes	Yes	Yes	Yes	Yes	Yes	Unclear	Yes	Yes	Yes	Low	Low	Yes
Sessa [22]	Yes	Yes	Yes	Yes	Yes	Yes	Unclear	Yes	Yes	Yes	Low	Low	Yes
Valentino [23]	No	No	Yes	Yes	Yes	Yes	Yes	Yes	Yes	Yes	High	Low	Yes
Valentino [24]	No	No	Yes	Yes	Yes	Yes	Unclear	Yes	Yes	Yes	High	Low	Yes

**Fig. 3:**
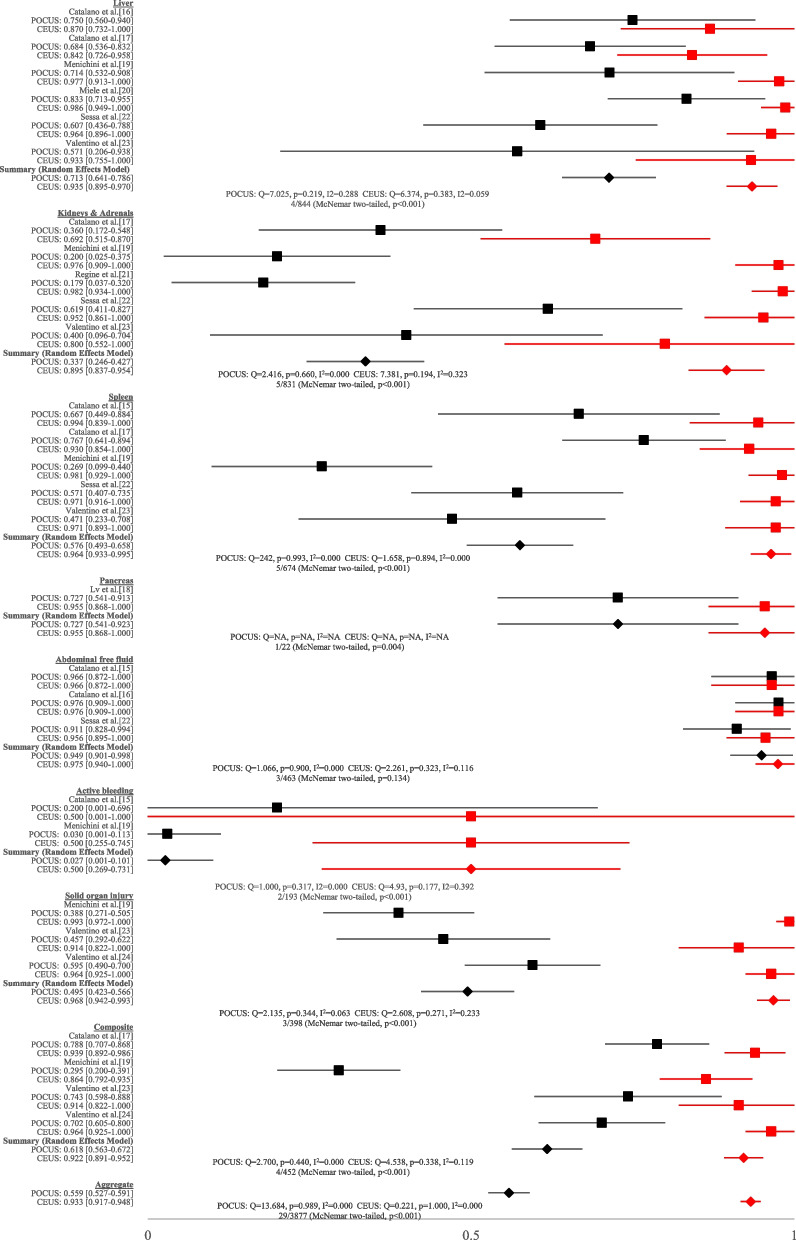
Forest plots of sensitivities of all studies that performed contrast-enhanced CT as the reference test. Black represents conventional POCUS. Red represents CEUS. n/N: number of studies/total diagnostic tests

**Fig. 4:**
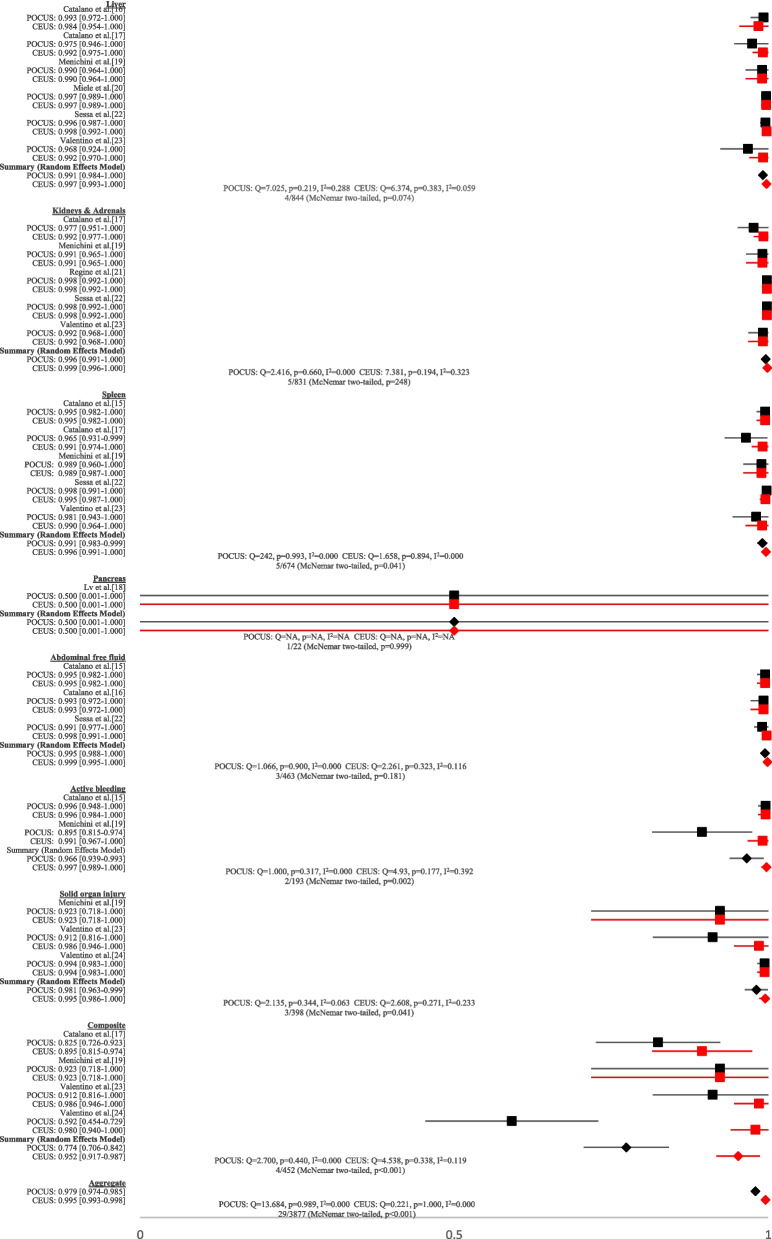
Forest plots of specificities of all studies that performed contrast-enhanced CT as the reference test. Black represents conventional POCUS. Red represents CEUS. n/N: number of studies/total diagnostic tests. NA: not available

A total of 30 pairwise comparisons between conventional ultrasound and CEUS using results from CT imaging as standard reference were used for subgroup analyses, producing a combined sample size of 3877. Forest plots of all studies are shown in Figs. 3 and 4. Sensitivity ranged from 0.030 to 0.976 for conventional ultrasound and 0.500 to 0.994 for CEUS. CEUS sensitivities for all individual studies were equal or superior to conventional ultrasound results. Specificity ranged from 0.500 to 0.998 for both modalities. CEUS specificities for all individual studies except for two [16, 22] were superior to their conventional ultrasound counterparts.

When paired datasets were examined, as presented in Figs. 3 and 4, we found that CEUS had significantly higher overall sensitivity of 0.933 (95% CI 0.917–0.948) versus 0.559 for conventional ultrasound (95% CI 0.527–0.591) (two-tailed, *P* < 0.001). The specificity of pooled datasets for CEUS of 0.995 (95% CI 0.993–0.998) was also significantly higher than conventional ultrasound at 0.979 (95% CI 0.974–0.985) (two-tailed, *P* < 0.001). Further subgroup analyses revealed that CEUS had statistically higher sensitivity when evaluating the liver (two-tailed, *P* < 0.001), kidneys and adrenals (two-tailed, *P* < 0.001), spleen (two-tailed, *P* < 0.001), pancreas (two-tailed, *P* < 0.01), the presence of active bleeding (two-tailed, *P* < 0.001), any solid organ injury (two-tailed, *P* < 0.001), and in the composite subgroup (two-tailed, *P* < 0.001) when compared to conventional ultrasound. When subgroup data was paired to conventional ultrasound, CEUS demonstrated statistically significant superiority with regards to specificity for the spleen (two-tailed, *P* < 0.05), the presence of active bleeding (two-tailed, *P* < 0.01), solid organ injury (two-tailed, *P* < 0.05), and in the composite subgroup (two-tailed, *P* < 0.001). While only one study yielded results for the sonographic examination of the pancreas [18], CEUS sensitivity was still significantly higher than conventional ultrasound.

The diagnostic odds ratio ranged from 21.0 to 29,568.0 for CEUS versus 0.8 to 285.3 when utilizing conventional ultrasound depending on the pathologic subgroup. When pooled, the diagnostic odds ratio for all noted pathologies carried an odds ratio of 3036.9 (95% CI 3036.3–3037.5) for CEUS in comparison to 60.4 (95% CI 60.1–60.7) for conventional ultrasound. Values are presented in Table 3.

**Table 3:** Pooled diagnostic odds ratio, number needed to diagnose, and likelihood ratios

	Pooled diagnostic odds ratio (95% CI)		Pooled Numbers needed to diagnose (95% CI)		Positive likelihood ratio (95% CI)		Negative likelihood ratio (95% CI)	
Conventional ultrasound								
OVERALL	60.4	(60.1–60.7)	1.297	(1.256–1.342)	20.55	(20.14–20.96)	0.41	(0.36–0.45)
Liver	285.3	(284.5–286.2)	1.126	(1.076–1.181)	82.51	(80.53–84.48)	0.29	(0.17–0.40)
Kidneys and adrenals	122.4	(121.2–123.6)	1.199	(1.086–1.338)	49.07	(47.34–52.80)	0.67	(0.61–0.72)
Spleen	143.7	(142.8–144.7)	1.189	(1.119–1.269)	51.41	(49.43–53.40)	0.43	(0.33–0.53)
Pancreas	2.7	([−1.4]–6.7)	21.450	([−2.674]–2.141)	1.45	(0.78–2.13)	0.55	([−0.80]–1.89)
Abdominal free fluid	92.0	(91.8–92.9)	1.038	(1.001–1.077)	14.68	(14.04–15.33)	0.21	(0.02–0.41)
Active bleeding	0.8	([−2.1]–3.7)	51.723	([−13.523]–8.880)	1.42	([−2.28]–5.12)	0.98	(0.84–1.13)
Solid organ injury	50.9	(49.9–51.9)	1.545	(1.388–1.743)	26.22	(24.77–27.66)	0.52	(0.40–0.63)
Composite	5.5	(5.1–6.0)	2.918	(2.368–3.801)	2.70	(2.44–2.96)	0.50	(0.32–0.67)
Contrast-enhanced ultrasound								
OVERALL	3036.9	(3036.3–3037.5)	1.038	(1.030–1.046)	125.68	(124.99–126.37)	0.07	([−0.04]–0.19)
Liver	4926.4	(4924.8–4927.9)	1.029	(1.009–1.049)	378.43	(375.38–381.47)	0.06	([−0.22]–0.34)
Kidneys and adrenals	6195.5	(6193.4–6197.5)	1.026	(1.005–1.048)	759.97	(745.46–765.47)	0.10	([−0.10]–0.30)
Spleen	7142.2	(7140.5–7143.9)	1.025	(1.004–1.046)	201.14	(199.07–203.21)	0.03	([−0.45]–0.50)
Pancreas	21.0	(16.6–25.4)	3.225	(1.89–11.013)	253.00	(246.44–259.56)	0.04	([−0.78]–0.86)
Abdominal free fluid	29,568.0	(29,564.9–29,571.1)	1.012	(0.994–1.030)	613.47	(606.97–619.98)	0.03	([−0.55]–0.62)
Active bleeding	350	(347.1–352.9)	1.113	(0.961–1.321)	733.62	(720.3–746.93)	0.35	(0.24–0.46)
Solid organ injury	6330	(6327.9–6332.1)	1.034	(1.022–1.047)	205.16	(203.04–207.28)	0.03	([−0.70]–0.76)
Composite	233.3	(232.5–234.2)	1.207	(1.172–1.244)	16.07	(15.61–16.53)	0.08	([−0.45] –0.61)

As shown in Table 3, the number needed to diagnose values for conventional ultrasound ranged from 1.038 to 51.723, in contrast to CEUS examinations that ranged from 1.012 to 3.225. A comparison of the two modalities revealed that two conventional ultrasound diagnostic categories (pancreas and the presence of active bleeding) were not statistically significant (two-tailed, *P* > 0.05), whereas all CEUS categories were statistically significant (two-tailed, *P* < 0.05).

The positive likelihood ratio ranged from 1.42 to 82.51 for conventional ultrasound POCUS in comparison to 16.07 to 759.97 for CEUS, while the negative likelihood ratio ranged from 0.19 to 0.98 for conventional ultrasound versus 0.03 to 0.35 for CEUS. Identification of pancreatic injury and the presence active bleeding were statistically non-significant (two-tailed *P* > 0.05) for conventional ultrasound imaging, whereas all all CEUS categories were statistically significant (two-tailed *P* > 0.05). Results are found in Table 3.

Using the random effects model, the Cochran’s Q for conventional ultrasound was 4.311 with 28 degrees of freedom (two tailed, *P* > 0.05) while for CEUS it was 0.221 with 28 degrees of freedom (two tailed, *P* > 0.05), indicating that the proportion of variability of measurements for both modalities are the same in the population when considering overall results. Subsequently, the Higgin’s I^2^ for overall results was 0.000 for conventional ultrasound and 0.000 for CEUS, indicating low heterogeneity for both methods. Similarly, subgroup analysis revealed low heterogeneity for each category of solid organ injury or finding with the exception of the pancreas, which had only one study available for analysis.

**Fig. 5:**
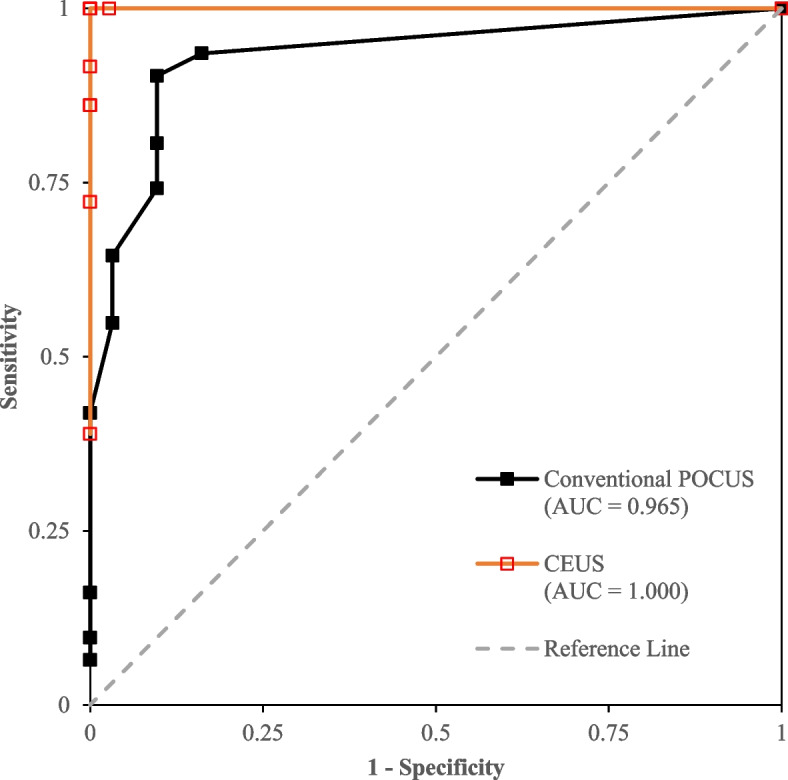
SROC curve plotting sensitivity (true positive rate) against 1-specificity (false positive rate)

Both conventional ultrasound and CEUS produced outstanding discriminating abilities for all categories, as shown in Fig. 5. The AUC for overall datasets was 0.965 for conventional ultrasound, whereas CEUS was marginally higher at 1.000.

## Discussion

To our knowledge, this is the first systematic review and meta-analysis examining and directly comparing the performance of contrast to conventional ultrasound diagnostic tests in abdominal trauma. While both modalities performed equally well with identifying abdominal free fluid, CEUS performed significantly better than conventional ultrasound at ruling out every solid organ injury of interest in abdominal trauma. Our results suggest that in a direct comparison, CEUS is superior to conventional ultrasound as the initial screening test of patients with abdominal trauma and raises the question as to whether it should be integrated as common practice when performing the FAST examination. One of the aims of this systematic review was to assess whether CEUS can be the next logical progression of clinical ultrasound in the abdominal trauma setting.

First, the diagnostic accuracy of CEUS is considerably greater to conventional ultrasound for traumatic abdominal injuries. Previous systematic reviews have shown that for patients with abdominal trauma, a negative FAST exam could not reliably rule out injury given its low sensitivity [25, 26] with the criticism that a FAST either provides false reassurance, leads to increased testing from borderline findings, or has no evidence based bearing on the decision to perform further diagnostic testing at all [26]. Our analysis produced similar results with an overall sensitivity of 0.559 for conventional ultrasound. In contrast, CEUS had an overall sensitivity that was significantly more robust at 0.933, indicating that it performs better at ruling out injury. Additionally, while both modalities exhibited nearly equivalent diagnostic accuracies for the identification of intraperitoneal free fluid, our systematic review established that active intraabdominal hemorrhage can only be appreciated on CEUS, a substantial advantage over conventional POCUS, which exhibited poor and nonsignificant values for diagnostic odds ratio, number needed to diagnose, and likelihood ratios. According to current literature, CEUS has the capability of identifying active bleeding in other locations as well, such as the thorax [27] and gastrointestinal tract [28]. We hope future studies further build on the data by including extraabdominal FAST-related findings of interest, specifically hemothoraces and hemopericardium to better understand the future role of CEUS in identifying active bleeding. Furthermore, the ability to visualize the extravasation of contrast in continuous or pulsatile form using CEUS [29] demonstrates great potential to dynamically characterize static CT findings. We postulate CEUS has additional monitoring and resuscitative clinical ultrasound roles in situations where an area of extravasation is identified with CT imaging, bringing with it an entirely new dimension to the FAST examination.

Second, utilization of CEUS during trauma resuscitation should achieve similar, if not better, survival outcomes than conventional ultrasound. The REACT-2 study, a large international, multicenter, randomized control trial published in the Lancet revealed no mortality benefit to performing an immediate total-body CT scan in patients with severe trauma than conducting standard radiological work-up, which included radiographs and FAST examination during primary survey, with selective CT scans following further assessment and resuscitation [30]. While our systematic review measured substantial benefits of using contrast for sonographic assessments, there are clear advantages of using CEUS over CT imaging in an emergency context as well: CEUS is rapidly available [29], and therefore primary surveys can be completed on multiple patients that will undoubtedly prove beneficial during mass trauma events; assessments are performed bedside, thereby eliminating the risk of transporting hemodynamically unstable patients to a radiology suite [28]; serial measurements allow for injury monitoring and follow-up [31]; and exposure to radiation is eliminated, thus avoiding potential risk to pregnant women and children [32]. Although a notable limitation is that CEUS does not allow for complete complete visualization of the whole abdomen, we believe the mortality benefits of restricting assessments to intraabdominal solid organs and spaces will outweigh immediate total-body CT scan during trauma resuscitation.

Third, trauma teams must have the confidence that there will be sufficient time to administer contrast agent prior to the FAST exam. For the included studies of our systematic review, all authors reported using CEUS as a part of or even in place of a FAST examination. During the evaluation of nine hemodynamically unstable patients, Catalano et al. [16] reported the room time for baseline conventional ultrasound and CEUS evaluation was, at maximum, 6 minutes. We believe that CEUS examination time can be optimized to occur concurrently during the trauma survey if timed appropriately. We do not think of CEUS as a separate procedure to the FAST, but that the use of contrast can be used as an adjunct to enhance the images. During the primary survey and resuscitation, once airway and breathing are established and intravenous access is obtained in the antecubital vein using an 18- or 20-gauge needle for adults, or an 18- to 24-gauge needle for pediatric patients, a dose of 1.2 to 4.8 mL ultrasound contrast will be administered, followed by intravenous fluid therapy and blood replacement if required. Enhancement will begin 10 seconds [29] following injection of the contrast, and will remain in the body for approximately 10 to 15 minutes [33]. This provides a sizable window of time during the initial trauma evaluation for the FAST study to be performed immediately following portable X-ray imaging while only requiring a flush of contrast during IV line placement. If the contrast has not sufficiently diffused the organs by the time an FAST is completed, or too much time has elapsed before an FAST can be performed, then the opportunity to use contrast is lost. As mentioned previously, most of the included studies used SonoVue as the contrast agent. Although it was shown to have a quicker transit time in the parenchyma than the first generation contrast agent Levovist (Schering) [34], no studies have directly compared the biomechanics of various contrast agents following traumatic abdominal injuries. Finally, possible CEUS scanning strategy may be to systematically explore abdominal solid organs and spaces on one side of the abdomen before continuing to the other, in as little 1 to 3 minutes, as accomplished by multiple included studies in our systematic review [16, 17, 19]. Theoretically, this will offer little temporal deviation from a standard FAST study and can occur concurrently while team members complete the secondary survey, perform interventions, and prep the patient for transport.

Our study, nonetheless, had few limitations of note. First, our study did not investigate allergic complications that may arise with CEUS. Nonetheless, although prior research has supported an excellent safety profile when used for cardiology applications compared to agents such as low osmotic iodinated intravenous contrast media [35], it is important to assess all patients for the presence of any condition that precludes administration, and always have anti-allergy therapy, resuscitation equipment, and trained personnel readily available [32]. Second, nearly half of our included studies had high risk patient selection bias. Since CEUS examination often followed a baseline assessment by conventional ultrasound, this partiality could have led to a potential overestimation of diagnostic performance of CEUS examinations [36]. Third, two of the ten included studies had sample sizes under 50, which could have led to an overestimated effect size as a result [37]. For example, only one study yielded data for the pancreas, which influenced the large confidence intervals for sensitivity and specificity analyses for both modalities. However, it is important to note that pancreas evaluation by CEUS examination, but not conventional ultrasound, generated strong and statistically significant diagnostic odds ratio, number needed to diagnose value, and likelihood ratios despite results yielded from one study only. This specific analysis illustrates that even sample sizes of 10 to 20 may be large enough to produce appreciable meta-analysis results. Fourth, diagnostic performance could have been overestimated as the majority of included studies were designed as single-center trials [38, 39]. This demands that future multi-center trials be pursued to fulfill a gap in research. Finally, there were no studies that satisfied our inclusion criteria beyond 2015 [19, 22]. This raises the possibility of publication bias, whereby studies with striking results get published than those with less striking, and therefore unpublished, results [40].

In conclusion, we showed that the diagnostic value of CEUS was greater than that of conventional ultrasound in the abdominal trauma work up. CEUS exhibited strong sensitivity, which makes it poised to be an answer to the criticisms that conventional FAST examination faces today. Future studies should investigate whether the incorporation of CEUS as an initial assessment tool is diagnostically superior to the conventional bedside FAST examination during the evaluation of abdominal trauma and whether it influences morbidity, mortality, or other patient-centered outcomes.

## Supplementary Information

**Additional file 1.** PRISMA 2020 Checklist.

**Additional file 2.** Equations.

## Data Availability

The datasets used and analyzed during the current study are available from the corresponding author on reasonable request.
