# Supplementary material for: Is it time to re-think FAST? A systematic review and meta-analysis of Contrast-Enhanced Ultrasound (CEUS) and conventional ultrasound for initial assessment of abdominal trauma
Source: BMC Emerg Med. 2023 Jan 27;23:8. doi: 10.1186/s12873-023-00771-4 (PMC9881326; doi:10.1186/s12873-023-00771-4)
Supplement: Supplementary file 2 — Additional file 2. Equations. [file 12873_2023_771_MOESM2_ESM.docx]

**Equations**


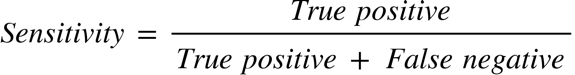


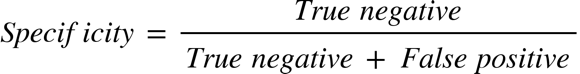


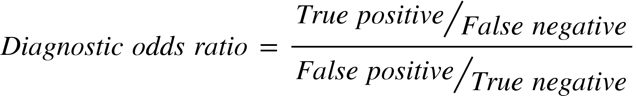


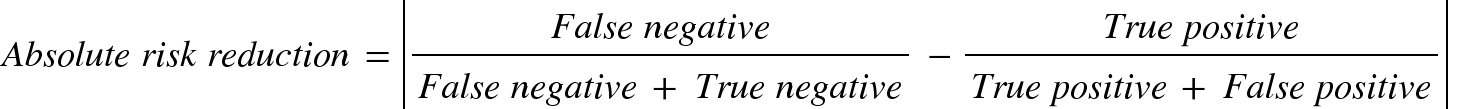


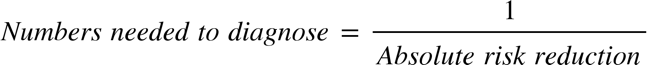


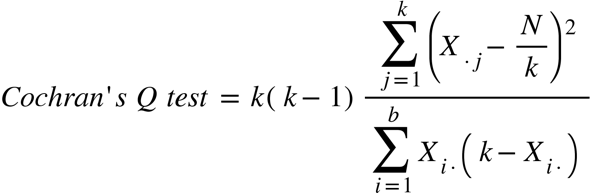


where

k is the number of treatments

X_.j_ is the column total for the j^th^ treatment

b is the number of blocks

X_i._ is the row total for the i^th^ block

N is the grand total


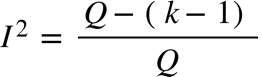


where

Q is Cochran's Q test


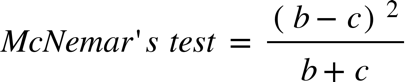


where

|  | Test 2 positive | Test 2 negative | Row total |
| --- | --- | --- | --- |
| Test 1 positive | a | b | a + d |
| Test 1 negative | c | d | c + d |
| Column total | a + c | b + d | N |
